# Supplementary material for: A molecular switch in sulfur metabolism to reduce arsenic and enrich selenium in rice grain
Source: Nat Commun. 2021 Mar 2;12:1392. doi: 10.1038/s41467-021-21282-5 (PMC7925690; doi:10.1038/s41467-021-21282-5)
Supplement: Supplementary file 1 — Supplementary Information [file 41467_2021_21282_MOESM1_ESM.pdf]

A molecular switch in sulfur metabolism to reduce arsenic and enrich selenium in rice grain

Sheng-Kai Sun[1], Xuejie Xu[1], Zhong Tang[1], Zhu Tang[1], Xin-Yuan Huang[1], Markus Wirtz[2], Rüdiger Hell[2], Fang-Jie Zhao[1*]

[1] State Key Laboratory of Crop Genetics and Germplasm Enhancement, College of Resources and Environmental Sciences, Nanjing Agricultural University, Nanjing 210095, China

[2] Centre for Organismal Studies (COS), Heidelberg University, 69120 Heidelberg, Germany

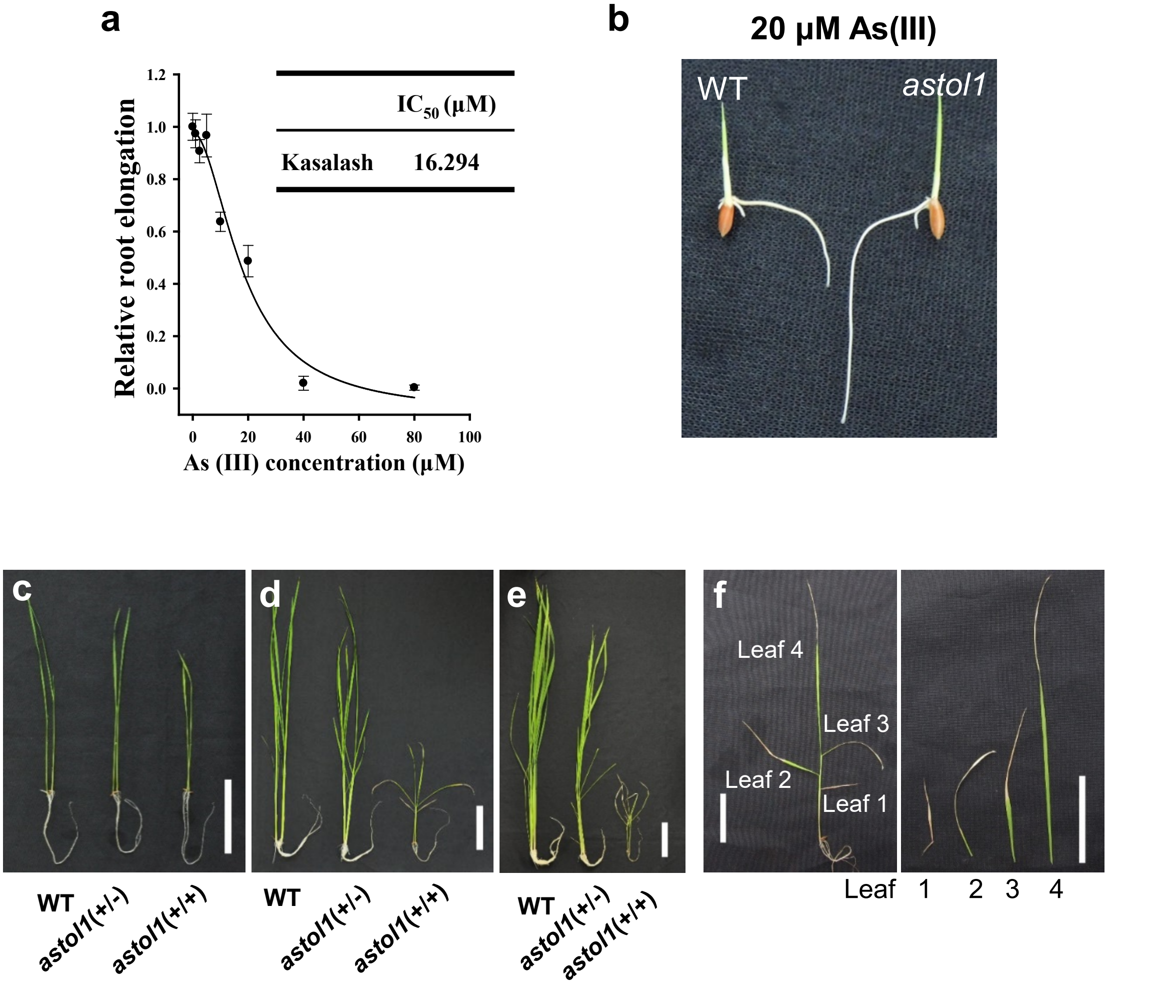

Supplementary Figure 1 The phenotype of *astol1* mutant.
(a) Relative root elongation of WT cv. Kasalath in different As(III) concentration. Date are shown as means ± s.d., n = 8 biological replicates for 5 μM As(III) treatment, 9 biological replicates for 10 μM As(III) treatment, 10 biological replicates for all other As(III) treatment; each biological replicate represents an individual plant. **(b)** Root elongation of *astol1* in the primary round screen. **(c-e)** Growth phenotype of WT, *astol1*(+/-) and *astol1*(+/+) grown in a nutrient solution for 2 weeks **(c)**, 4 weeks **(d)** and 6 weeks **(e).** Two plants of each genotype are shown. Scale bars, 10 cm. **(f)** Phenotype of the whole plant of *astol1*(+/+) and the individual leaf (1–4 from older to younger). Scale bars, 5 cm. WT, wild type. *astol1*(+/-), *astol1* heterozygote. *astol1*(+/+), *astol1* homozygote.

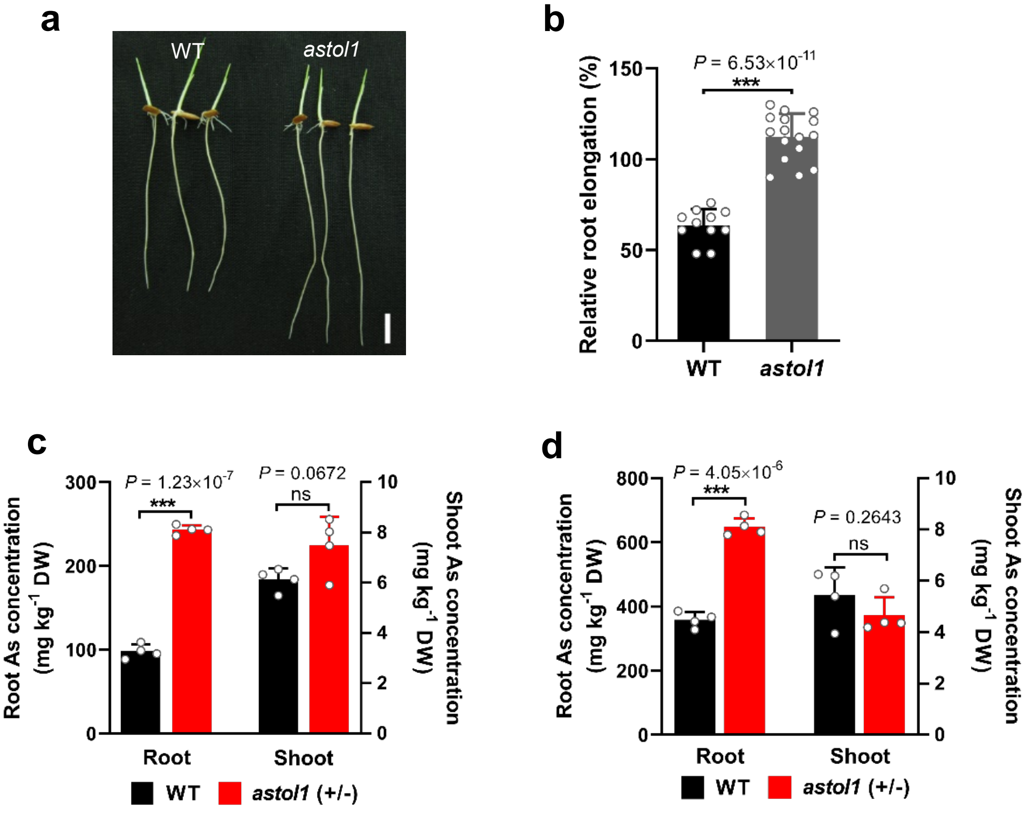

Supplementary Figure 2 The *astol1* mutant shows enhanced As(V) tolerance and higher As accumulation in the roots.

(a) Root elongation of wild-type and *astol1* plants treated with 8 μM As(V) for 2 days. Three plants each are shown. Scale bars, 10 mm. **(b)** Relative roots elongation of WT plants and *astol1* mutant treated with 8 μM As(V) for 2 days. **(c, d)** As concentrations in the roots and shoots of four-week-old WT plants and *astol1*(+/-) mutant after exposure to 5 μM As(III) (**c**) or 5 μM As(V) (**d**) for 3 days. DW, dry weight. WT, wild type. *astol1*(+/-), *astol1* heterozygote. Data in **b-d** are shown as means ± s.d., n = 11 for WT, 16 for *astol1* (**b**) or 4 (**c, d**) biological replicates; each biological replicate represents an individual plant. Asterisks in **b-d** indicate significant differences by two-sided Student's t-test: ***P<0.001. The letters "ns" indicate no significant differences.

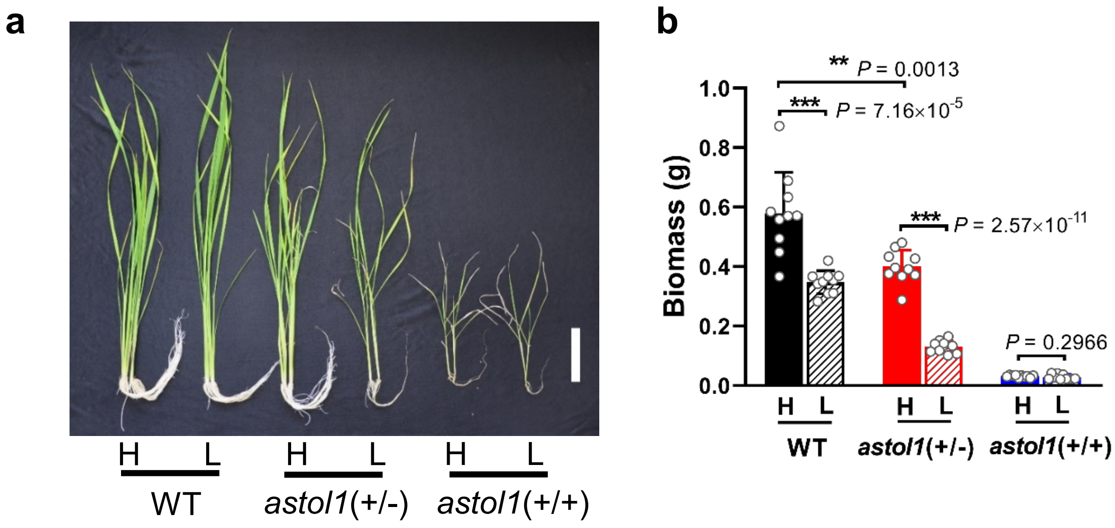

Supplementary Figure 3 Growth phenotype of wild type and *astol1* mutants grown hydroponically under different light intensity.

(a, b) Growth phenotype (**a**) and biomass (**b**) of 5-week-old WT, *astol1*(+/-) and *astol1*(+/+) plants grown hydroponically under different light intensity. H: High light (~800 μmol m^{-2} s^{-1}); L: Low light (~300 μmol m^{-2} s^{-1}). Two plants each are shown in **a**. Scale bars, 10 cm. WT, wild type. *astol1*(+/-), *astol1* heterozygote. *astol1*(+/+), *astol1* homozygote. Data in **b** are shown as means ± s.d., n = 10 biological replicates; each biological replicate represents an individual plant. Asterisks in **b** indicate the significant differences by two-sided Student's t-test: **P<0.01, ***P<0.001.

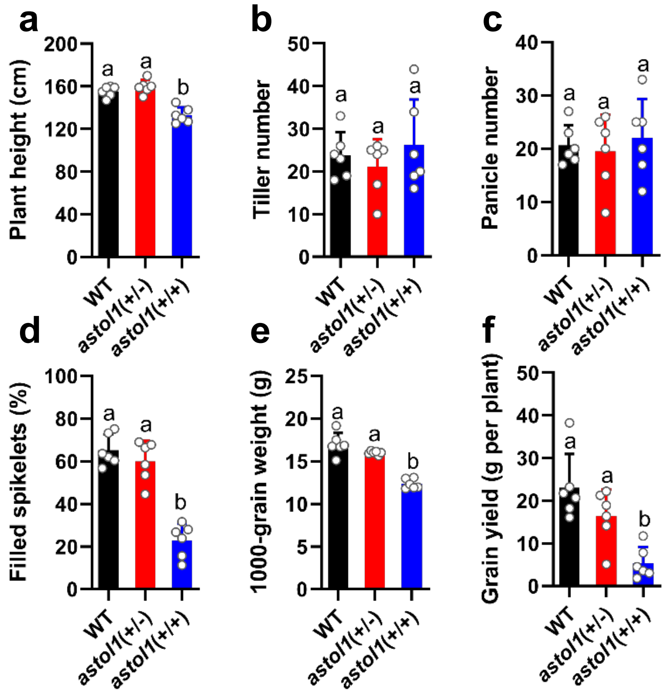

Supplementary Figure 4 Agronomic traits of wild type and *astol1* mutants grown in a paddy field. **(a-f)** Wild-type rice and *astol1* mutants were grown in a paddy field (Lingshui) until maturity. At harvest, plant height (**a**), tiller number (**b**) and panicle number (**c**) were recorded. After harvest, filled spikelets (**d**), 1000-grain weight (**e**) and grain yield (**f**) were recorded. WT, wild type. *astol1*(+/-), *astol1* heterozygote. *astol1*(+/+), *astol1* homozygote. Data are shown as means ± s.d., n = 6 biological replicates; each biological replicate represents an individual plant. Different letters in **a-f** indicate significant differences (P < 0.05) using one-way ANOVA followed by Tukey's test.

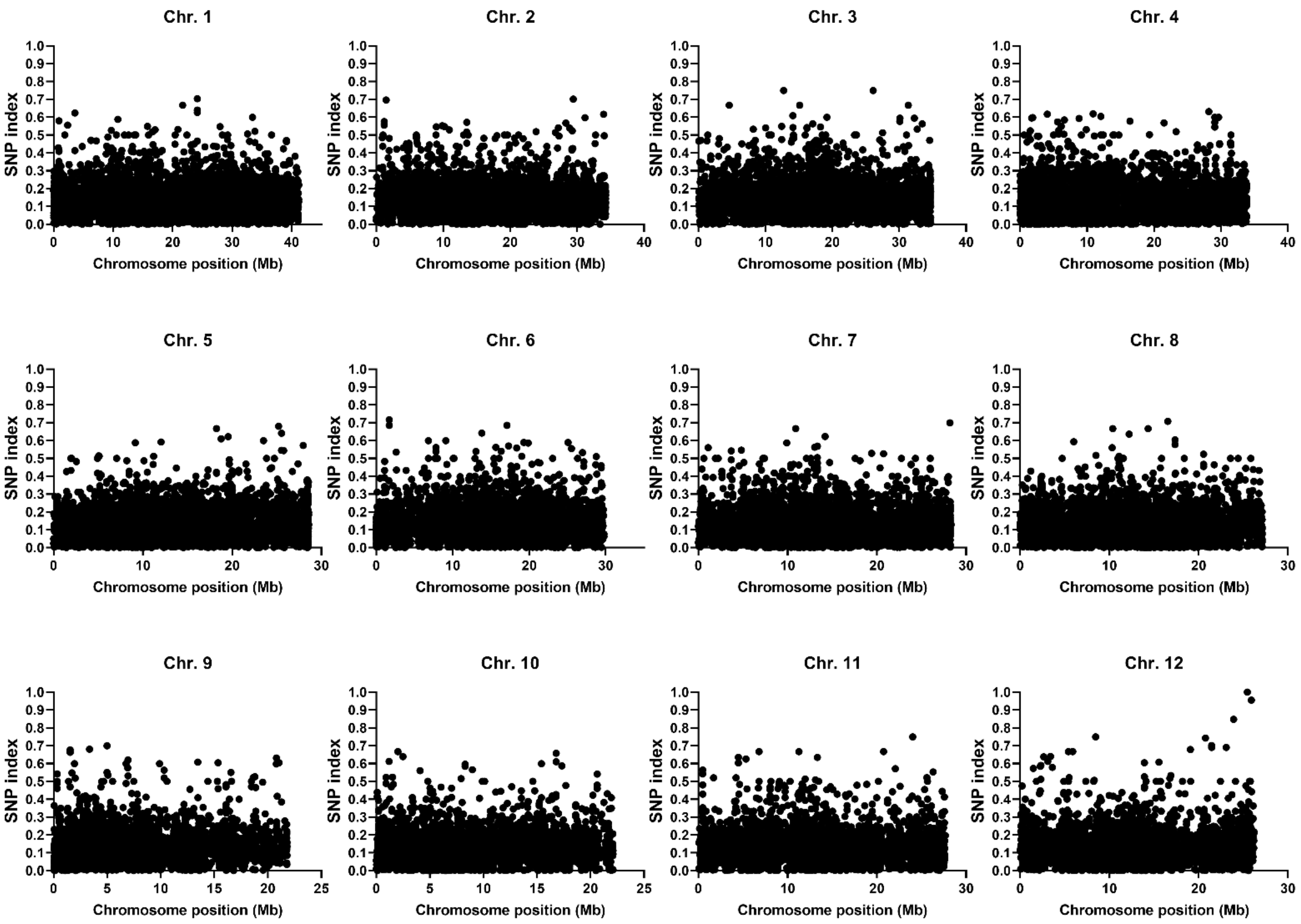

Supplementary Figure 5 SNP index plots for 12 chromosomes of the *astol1*(+/+) mutant.
SNP index plots for the *astol1*(+/+) mutant showing chromosomes 1-12, respectively. Black circles indicate SNP positions and their SNP indices.

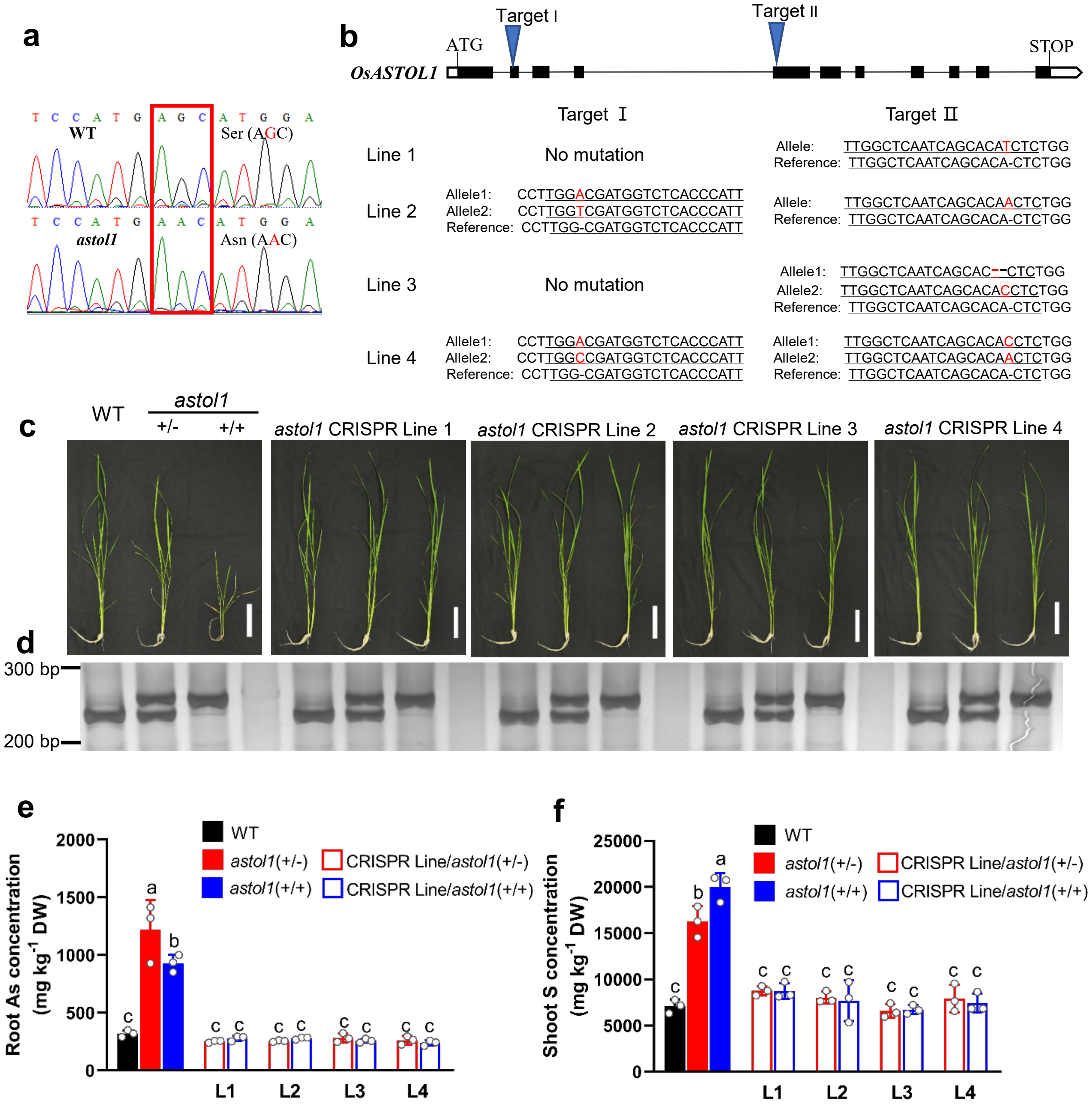

Supplementary Figure 6 Rescue of the mutant phenotype by knock-out of *Osastol1* in *astol1*.
(a) Confirmation of the *astol1* mutation by sequencing. The G-to-A mutation is shown inside the red box. **(b)** Sketch map of mutations of CRISPR knockout lines in the *astol1*(+/-) background. **(c)** Growth phenotype of five-week-old WT, *astol1*(+/-) and *astol1*(+/+) and CRISPR lines in the WT, *astol1*(+/-) or *astol1*(+/+) background. Two plants each are shown. Scale bars, 10 cm. **(d)** Confirmation of the genotype of plants in (**c**) by using the dCAPS marker targeted to the mutated site of *OSASTOL1*^{S189N} in *astol1*. Three times independent genotyping were shown similar results to the image in **d**. **(e, f)** Root As concentration (**e**) and shoot S concentration (**f**) of five-week-old WT, *astol1*(+/-), *astol1*(+/+), and CRISPR lines in the *astol1*(+/-) or *astol1*(+/+) background. Plants were grown hydroponically for five weeks, and then treated with 5 μM As(**III**) for 3 days. DW, dry weight. WT, wild type. *astol1*(+/-), *astol1* heterozygote. *astol1*(+/+), *astol1* homozygote. Data in **e, f** are shown as mean ± s.d., $n = 3$ biological replicates; each biological replicate represents an individual plant. Different letters in **e**, **f** indicate significant differences ($P < 0.05$) using one-way ANOVA followed by Tukey's test.

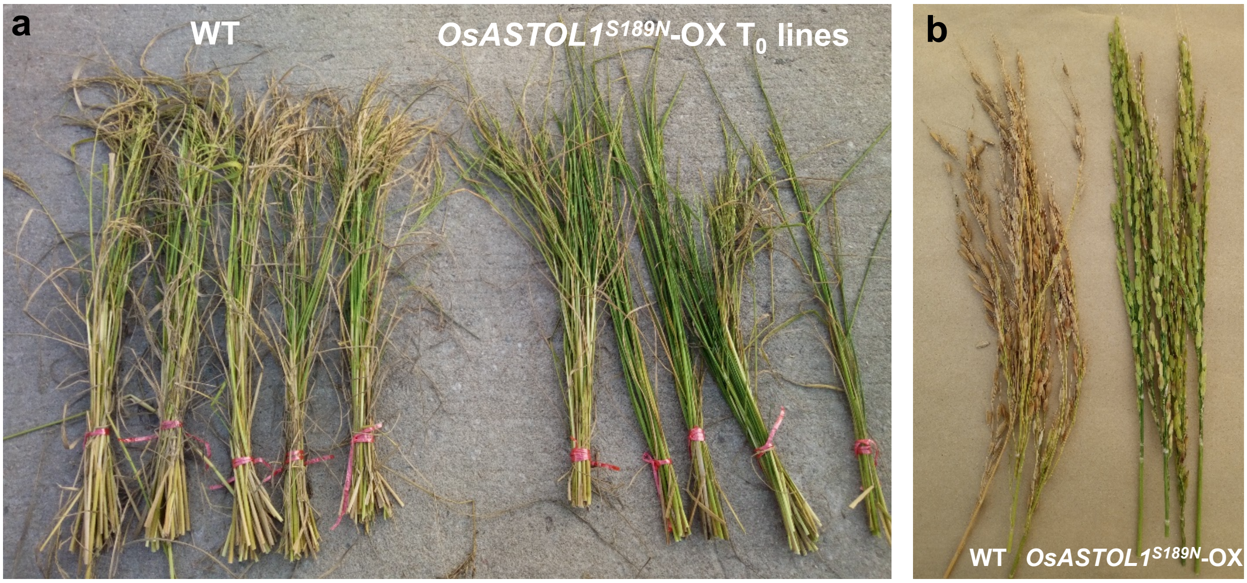

Supplementary Figure 7 Phenotype of wild type and *OsASTOL1*^{S189N}-OX T_0 transgenic lines grown in a paddy field.

(a) Wild-type (WT) plants and *OsASTOL1*^{S189N}-OX T_0 transgenic lines were grown in a paddy field (Lingshui) until maturity. **(b)** Panicles of WT and *OsASTOL1*^{S189N}-OX T_0 transgenic lines.

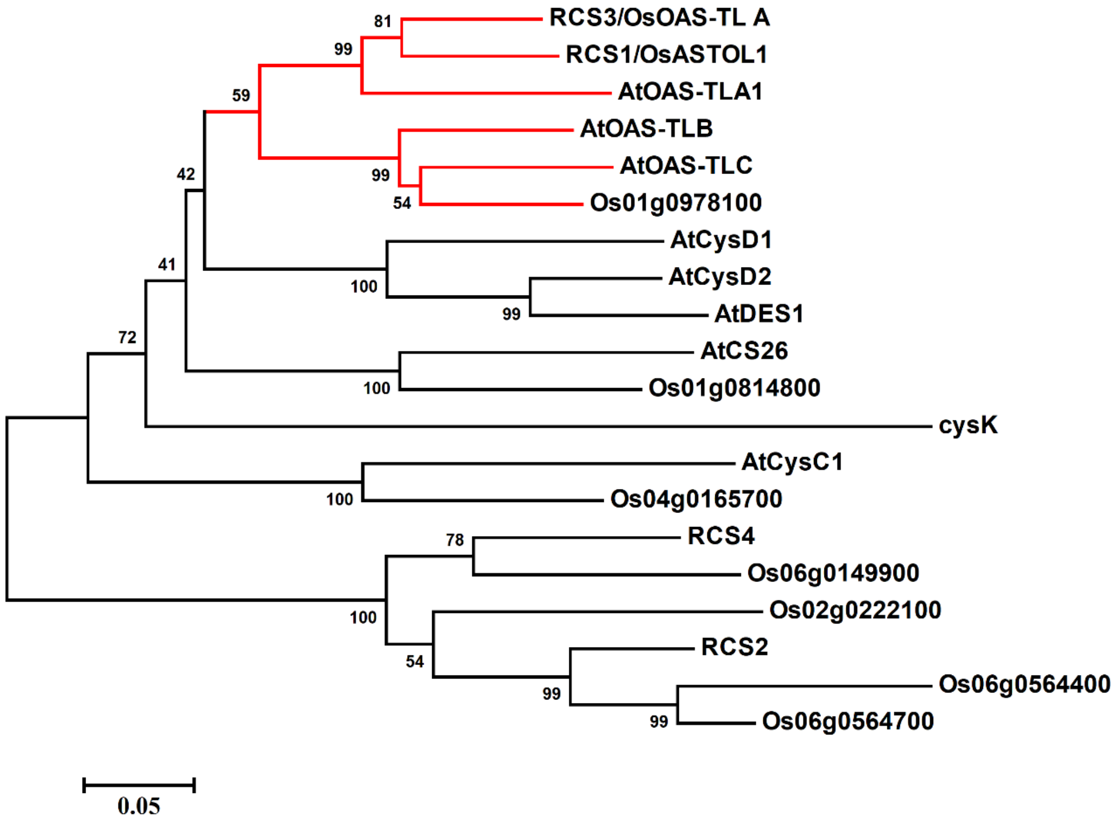

Supplementary Figure 8 Phylogenetic analysis of cysteine synthases in rice, *Arabidopsis thaliana* and *Escherichia coli*.

The phylogenetic tree was constructed using MEGA 4.0 software by Neighbor-Jointing method with 1000 bootstrapping trials. The numbers on the branch indicate bootstrapping values. AtOAS-TL A1: AT4G14880; RCS3/OsOAS-TL A1: Os03g0747800; RCS1/OsASTOL1: Os12g0625000; AtOAS-TL B: AT2G43750; AtOAS-TL C: AT3G59760; AtCysD1: AT3G04940; AtCysD2: AT5G28020; AtCS26: AT3G03630; AtCysC1: AT3G61440; AtDES1: AT5G28030; RCS2: Os06g0564500; RCS4: Os06g0149700; cysK: VWQ04957.

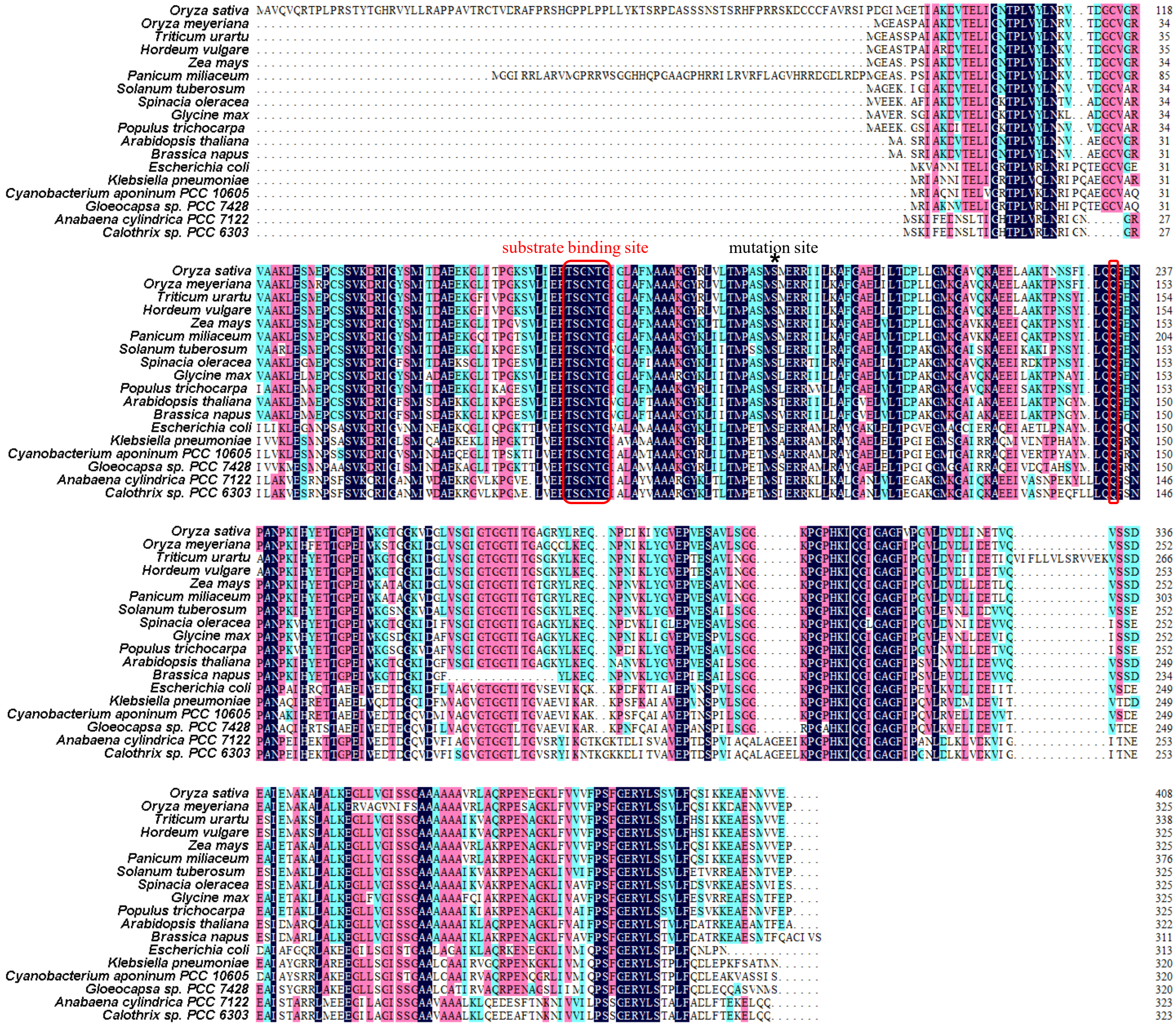

Supplementary Figure 9 Sequence alignment of cysteine synthase proteins.
Alignment of OsASTOL1 and cysteine synthase proteins using DNAMAN software. Red boxes and asterisk represent substrate binding sites and the mutation site, respectively. Accession numbers for aligned sequences: *Oryza sativa*: OsASTOL1, XP_015620529; *Oryza meyeriana*: AXS63115; *Triticum Urartu*: EMS62090; *Hordeum vulgare*: KAE8798328; *Zea mays*: NP_001105469; *Panicum miliaceum*: RLN28996; *Solanum tuberosum*: BAB20861; *Spinacia oleracea*: XP_021848847*; Glycine max*: NP_001235628; *Populus trichocarpa*: XP_006389317; *Arabidopsis thaliana*: AtOAS-TL A1, NP_001190732; *Brassica napus*: CDY09039; *Escherichia coli*: cysK, VWQ04957; *Klebsiella pneumoniae*: CDO15723; *Cyanobacterium aponinum PCC 10605*: AFZ54981; *Gloeocapsa sp. PCC 7428*: AFZ32243; *Anabaena cylindrica PCC 7122*: AFZ58860; *Calothrix sp. PCC 6303*: AFZ03783.

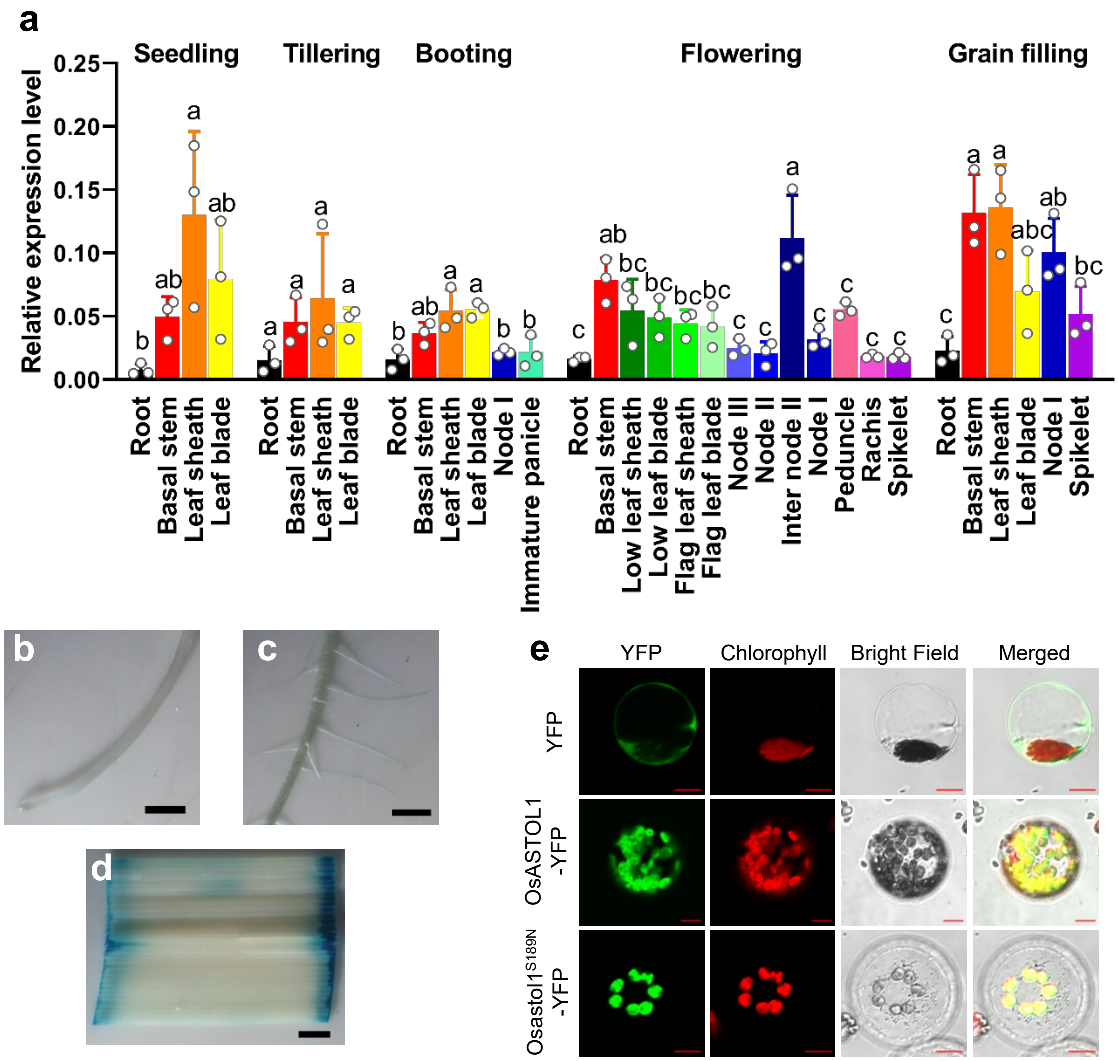

Supplementary Figure 10 Tissue expression pattern of *OsASTOL1* and subcellular localization of OsASTOL1.

(a) Tissue expression pattern of *OsASTOL1*. Samples of various organs from wild-type rice cv. Nipponbare grown in a paddy field (Nanjing) at different growth stages were collected for RNA extraction and Q-PCR analysis. *OsHistone H3* was used as the internal reference gene. Data are shown as mean ± s.d., $n = 3$ biological replicates; each biological replicate represents an individual plant. Different letters indicate significant differences ($P < 0.05$) using one-way ANOVA followed by Tukey's test. **(b-d)** *OsASTOL1* promoter activity in root tip (**b**), root mature zone (**c**) and leaf (**d**) as revealed by GUS staining. Scale bars, 500 µm. **(e)** Subcellular localization of OsASTOL1 in tobacco cells. Protoplasts were isolated from tobacco (*N. benthamiana*) leaves expressing eYFP (top), OsASTOL1-YFP (middle) or OsASTOL1^{S189N}-YFP (bottom) driven by the cauliflower mosaic virus 35S promoter. Left to right: YFP fluorescence, chlorophyll autofluorescence, bright-field images and merged images. Scale bars,10 µm. At least three independent transgenic lines or protoplasts were investigated and showed similar results to the images in **b-e**.

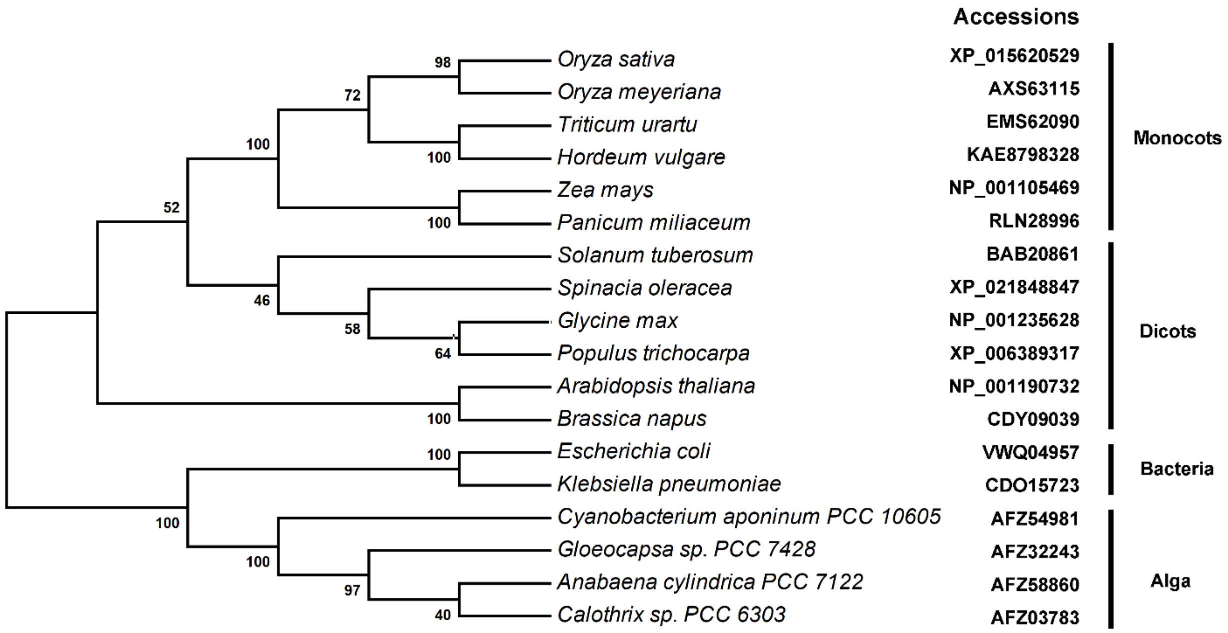

Supplementary Figure 11 Phylogenetic analysis of OsASTOL1 and homologous cysteine synthase proteins in various species from bacteria to plants.

The phylogenetic tree was constructed using MEGA 4.0 software by Neighbor-Jointing method with 1000 bootstrapping trials. The numbers on the branch indicate bootstrapping values. The accession numbers of OsASTOL1 and cysteine synthase proteins are shown at the right.

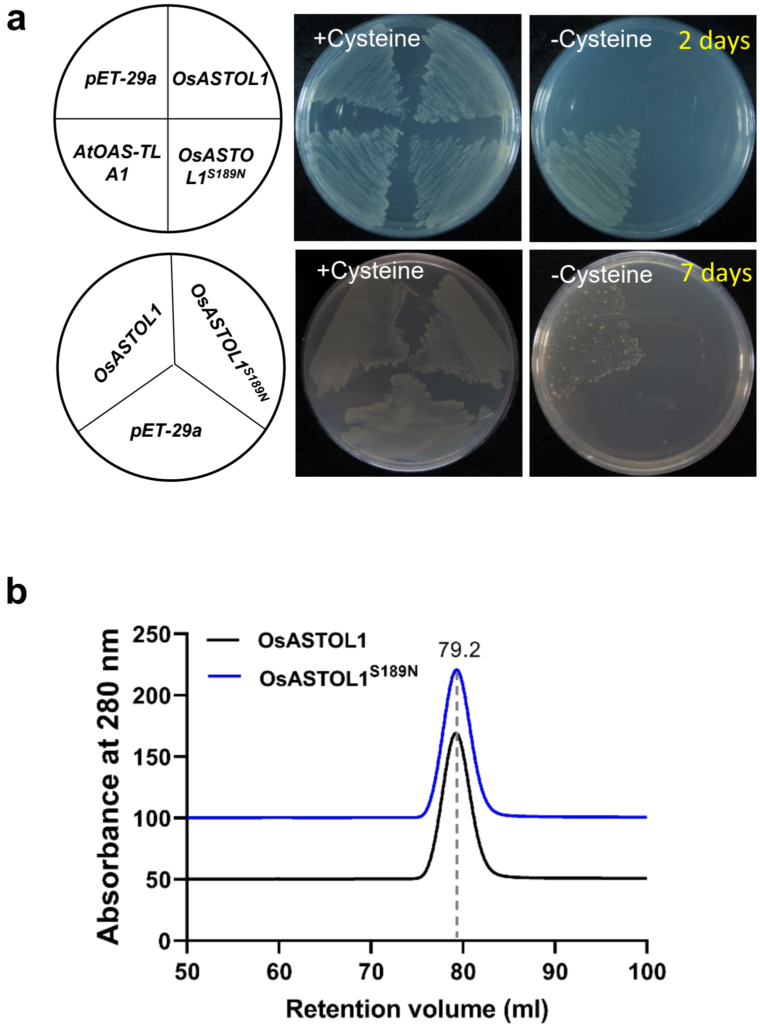

Supplementary Figure 12 The expression and genetic complementation of OsASTOL1 in *Escherichia coli.*

(a) Genetic complementation of OsASTOL1 in *E. coli*. The empty vector (pET-29a) and vectors harboring wild-type *OsASTOL1* and mutant *Osastol1* were transformed into the cysteine auxotroph *E. coli* NK3 strain. *AtOAS-TL A* was used as a positive control. All bacterial cells were grown on M9 media with or without 0.5 mM cysteine for 2 or 7 days. **(b)** Analysis of the molecular weight (MW) of purified OsASTOL1 and OsASTOL1^{S189N} proteins. 1 mg purified OsASTOL1 or OsASTOL1^{S189N} was loaded to size exclusion chromatography (SEC) using a Pharmacia FPLC^{TM} system. Both proteins eluted at the same retention volume (79.2 ml), which corresponds to a molecular size of 66.9 kDa, representing the OAS-TL dimer (calculated MW of an OsASTOL1 monomer is 33.8 kDa).

a

b

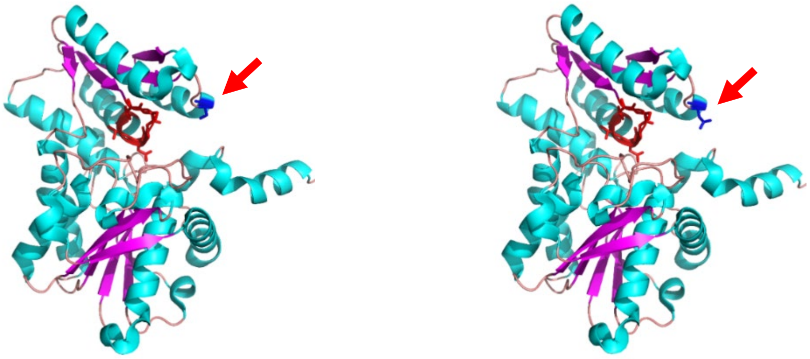

Supplementary Figure 13 Crystal structure of AtOAS-TL A protein

(a, b) Three-dimensional structure of AtOAS-TL A (PDB ID: 1z7w) (**a**) and a model of the AtOAS-TL A^{S102N} mutant protein generated with the PyMOL software (**b**). The substrate binding site and the mutation site are shown in red and blue, respectively. The arrows indicate the 102th amino acid residue in both proteins.

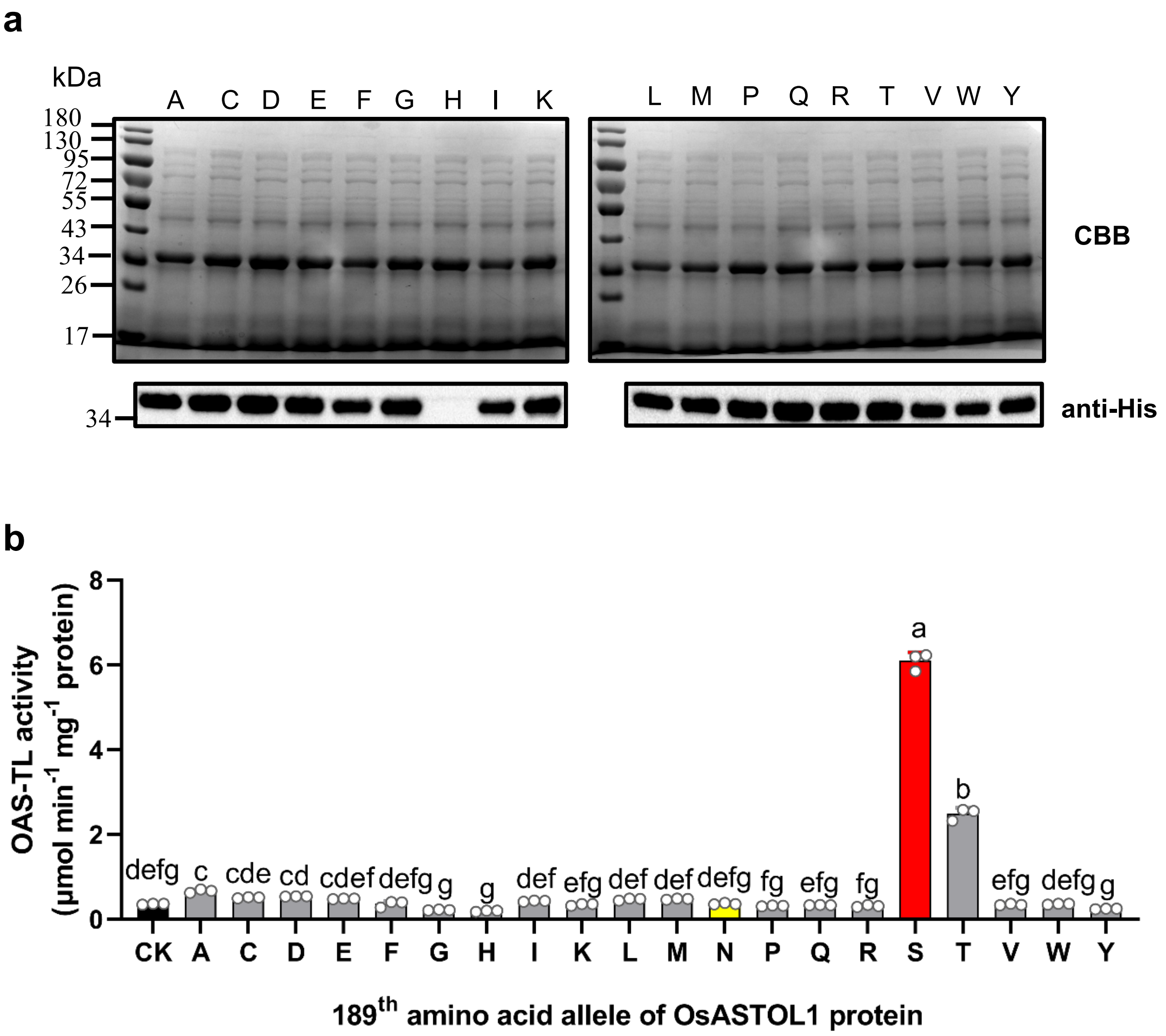

Supplementary Figure 14 ***In vitro*** **functional analysis of different allelic OsASTOL1 in** ***Escherichia coli.***

(a) The expression of different recombinant mature OsASTOL1 allele proteins in *E. coli*. Proteins were separated on SDS-PAGE and visualized by Coomassie Brilliant Blue (CBB) staining (upper) and by western blot with anti-His antibody (bottom). The experiment was repeated two times and similar results were obtained. **(b)** The OAS-TL enzyme activity in the crude extract of *E. coli* cells expressing different alleles of recombinant mature OsASTOL1 proteins. CK: empty vector; A: OsASTOL1^{S189A}; C: OsASTOL1^{S189C}; D: OsASTOL1^{S189D}; E: OsASTOL1^{S189E}; F: OsASTOL1^{S189F}; G: OsASTOL1^{S189G}; H: OsASTOL1^{S189H}; I: OsASTOL1^{S189I}; K: OsASTOL1^{S189K}; L: OsASTOL1^{S189L}; M: OsASTOL1^{S189M}; N: OsASTOL1^{S189N}; P: OsASTOL1^{S189P}; Q: OsASTOL1^{S189Q}; R: OsASTOL1^{S189R}; S: OsASTOL1^{S189} (wild-type); T: OsASTOL1^{S189T}; V: OsASTOL1^{S189V}; W: OsASTOL1^{S189W}; Y: OsASTOL1^{S189Y}. Data are shown as means ± s.d., n = 3 technical replicates. Different letters indicate significant differences (P < 0.05) using one-way ANOVA followed by Tukey's test.

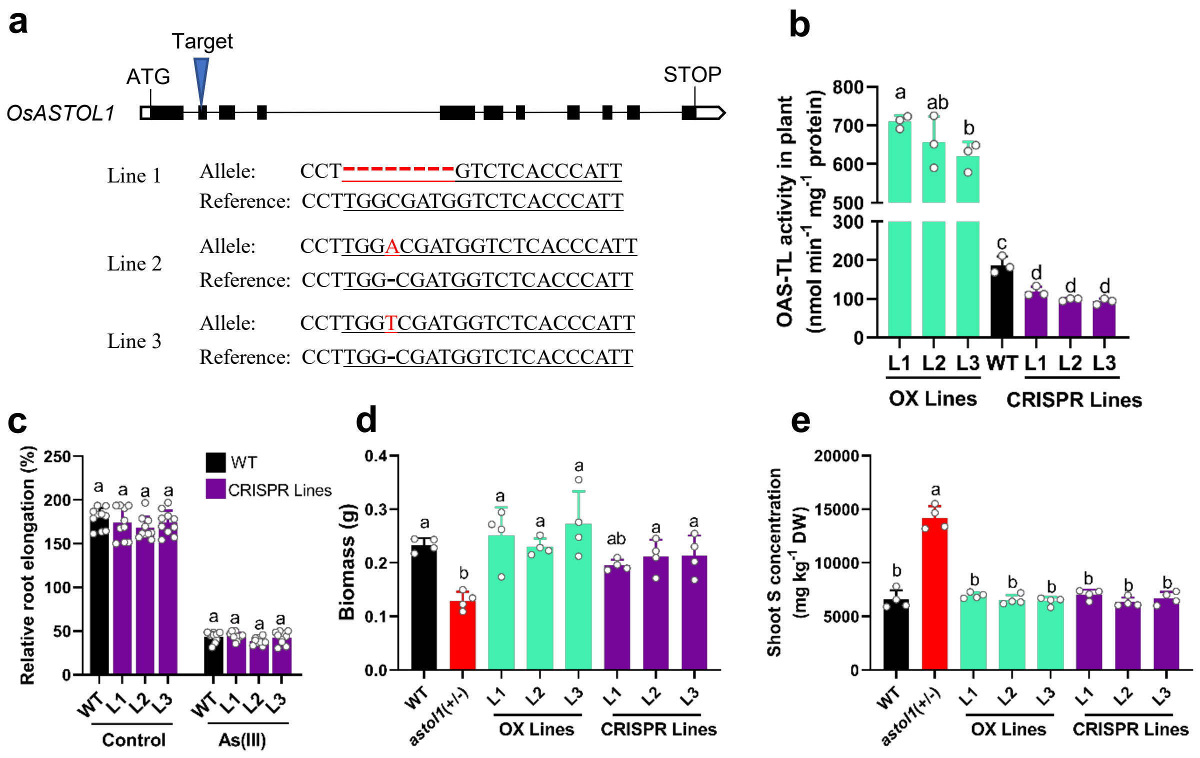

Supplementary Figure 15 The OAS-TL activity of OsASTOL1 is irrelevant to the phenotype of *astol1*. **(a)** Sketch map of mutations of CRISPR knockout lines in the *astol1*(+/-) background. (**b**) Total OAS-TL enzyme activity in the whole protein extracts of shoots of three-week-old WT, three independent overexpression (OX) lines and CRISPR lines in the WT background. (**c**) Relative root elongation of WT and three independent CRISPR lines (in the WT background) treated with 0 or 20 μM As(III) for 2 days. **(d, e)** Biomass (**d**) and shoot S concentration (**e**) of five-week-old WT, *astol1*(+/-), three independent *OsASTOL1*-overexpression lines (WT background) and three independent CRISPR lines (WT background). DW, dry weight. WT, wild type. *astol1*(+/-), *astol1* heterozygote. Data in **b-e** are shown as means ± s.d., $n = 3$ (**b**), 10 (**c**) or 4 (**d, e**) biological replicates; each biological replicate represents an individual plant. Different letters in **b-e** indicate significant differences ($P < 0.05$) using one-way ANOVA followed by Tukey's test.

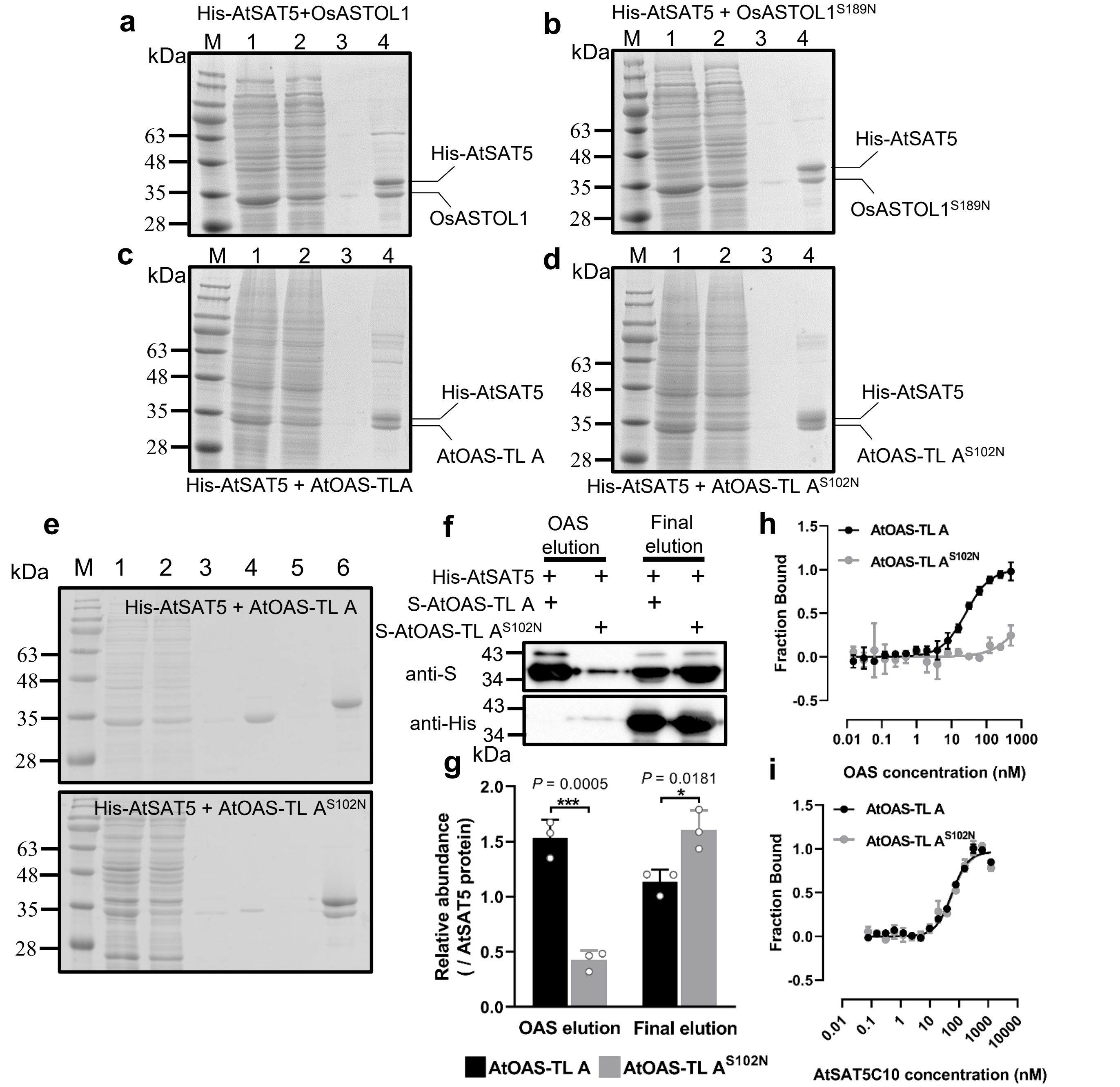

Supplementary Figure 16 ***In vitro*** **affinity analysis of OAS-TL proteins to SAT protein and OAS.**

(a-d) *In vitro* pull-down analysis of protein-protein interaction of OAS-TL proteins and AtSAT5 protein. The fraction of every step (lanes 1-4) from His-AtSAT5 and OsASTOL1 protein (**a**), His-AtSAT5 and OsASTOL1^{S189N} protein (**b**), His-AtSAT5 and AtOAS-TL A protein (**c**) and His-AtSAT5 and AtOAS-TL A^{S102N} protein (**d**) was analyzed by SDS-PAGE and visualized by Coomassie Brilliant Blue staining. M: marker; lane 1: crude extract of OsASTOL1 protein (**a**), OsASTOL1^{S189N} protein (**b**), AtOAS-TL A protein (**c**), and AtOAS-TL A^{S102N} protein (**d**); lane 2: flow through of OsASTOL1 protein (**a**), OsASTOL1^{S189N} protein (**b**), AtOAS-TL A protein (**c**), and AtOAS-TL A^{S102N} protein (**d**); lane 3: washing buffer (80 mM imidazol); lane 4: final elution (400 mM imidazol). The experiment was repeated two times and similar results were obtained. **(e)** *In vitro* pull-down analysis of the dissociation effect of OAS on CSC. The fraction of every step (lanes 1-6) from His-SAT5 and AtOAS-TL A protein (upper panel) and His-SAT5 and AtOAS-TL A^{S102N} protein (bottom panel) was analyzed by SDS-PAGE and visualized by Coomassie Brilliant Blue staining. M: marker; lane 1: crude extract of AtOAS-TL A protein (upper), and AtOAS-TL A^{S102N} protein (bottom); lane 2: flow through of AtOAS-TL A protein (upper), and AtOAS-TL A^{S102N} protein (bottom); lane 3: washing buffer (80 mM imidazol); lane 4: OAS elution (10 mM OAS+80 mM imidazol); lane 5: washing buffer (80 mM imidazol); lane 6: final elution (400 mM imidazol). The results were repeated three times and similar results were obtained. **(f)** the fraction of OAS elution and final elution was analyzed by SDS-PAGE and visualized by western blot, and a representative result of three repeated tests is shown. The experiment was repeated three times and similar results were obtained. **(g)** The relative abundance of AtOAS-TL A or AtOAS-TL A^{S102N} protein in the OAS elution and the final elution was quantified by ImageJ software, compared to corresponding AtSAT5 protein. **(h, i)** *In vitro* binding analysis of AtOAS-TL A and AtOAS-TL A^{S102N} protein with OAS (**h**) or AtSAT5C10 peptide (**i**). Data in **g-i** are shown as means ± s.d., n = 3 technical replicates. Asterisks in **g** indicate significant differences by two-sided Student's t-test: *P<0.05, ***P<0.001.

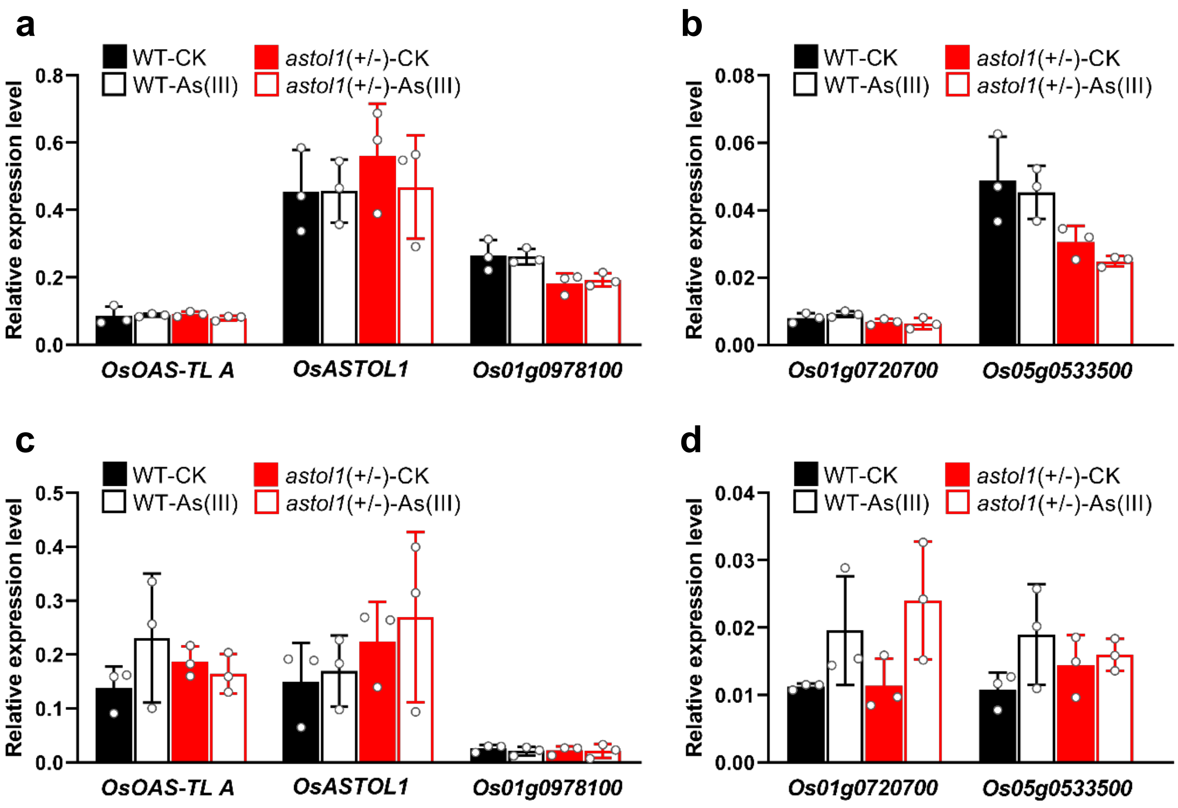

Supplementary Figure 17 The expression levels of *OsOAS-TL* and *OsSAT* genes in wild type and *astol1*(+/-) mutant.

(**a-b**) Relative expression levels of rice *OsOAS-TL* genes (**a**) and *OsSAT* genes (**b**) in the shoots of four-week-old WT and *astol1*(+/-) plants treated with or without 5 μM As(**III**) for 6 h. (**c-d**) Relative expression levels of rice *OsOAS-TL* genes (**c**) and *OsSAT* genes (**d**) in the roots of four-week-old WT and *astol1*(+/-) plants treated with or without 5 μM As(**III**) for 6 h. The transcripts were measured by Q-PCR, with *OsHistone H3* as the internal reference gene. WT, wild type. *astol1*(+/-), *astol1* heterozygote. CK, control. Data in **a-d** are shown as means ± s.d., *n* = 3 biological replicates; each biological replicate represents an individual plant.

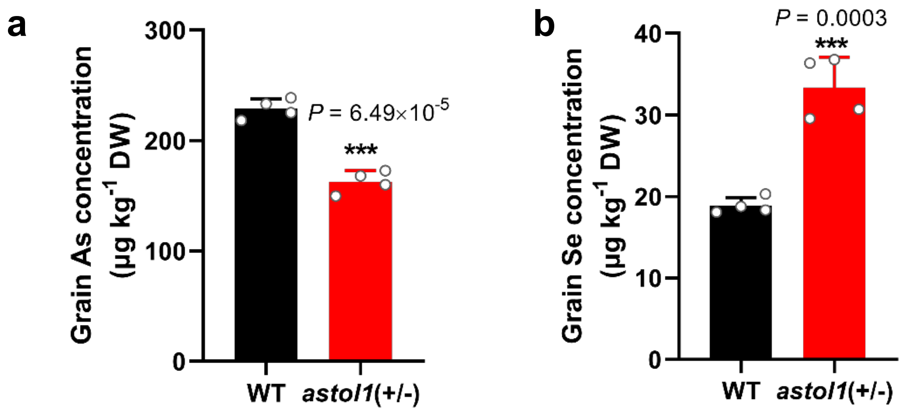

Supplementary Figure 18 The grain As and Se concentrations of wild type and *astol1* mutant.
(a) The As concentrations in rice grain of WT and *astol1*(+/-) plants grown in a paddy field (Lingshui). (**b**) The Se concentrations in rice grain of WT and *astol1*(+/-) plants grown in a paddy field (Lingshui). WT, wild type. *astol1*(+/-), *astol1* heterozygote. Data in **a, b** are shown as means ± s.d., n = 4 biological replicates; each biological replicate represents an individual plant. Asterisks in **a, b** indicate significant differences by two-sided Student's t-test: ***P<0.001.

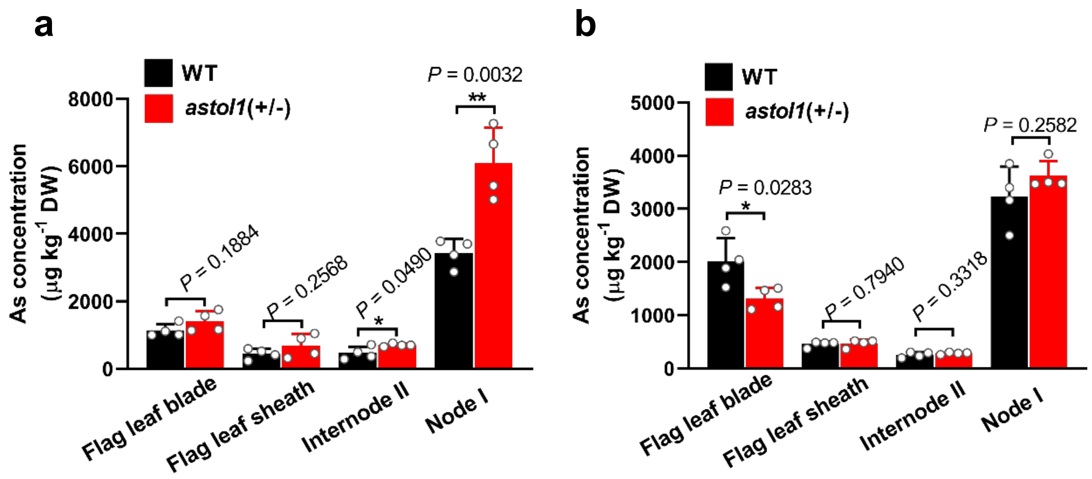

Supplementary Figure 19 The As concentrations in different tissues of wild type and *astol1* mutant at plant maturity.

(**a-b**) The As concentrations in different tissues of WT and *astol1*(+/-) mature plants grown in a paddy field at Lingshui (**a**) or Nanjing (**b**). WT, wild type. *astol1*(+/-), *astol1* heterozygote. Data in **a, b** are shown as means ± s.d., n = 4 biological replicates; each biological replicate represents an individual plant. Asterisks in **a, b** indicate significant differences by two-sided Student's t-test: *P<0.05, **P<0.01.

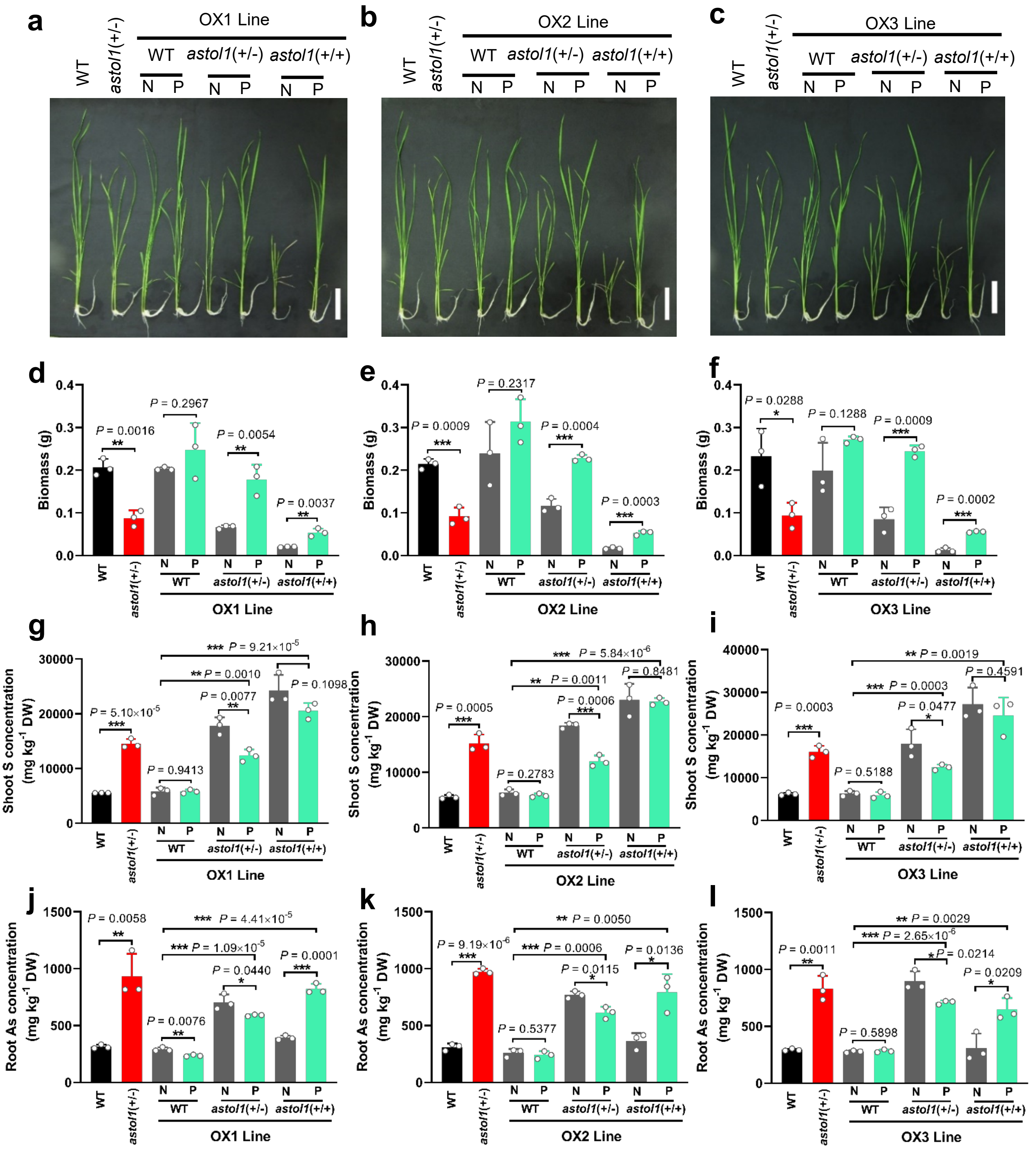

Supplementary Figure 20 Phenotypes of the *OsASTOL1* overexpression lines in the *astol1*(+/-) or *astol1*(+/+) background.
(a-c) Growth phenotype of five-week-old wild type (WT), *astol1*(+/-) and the overexpression line 1 (**a**), line 2 (**b**) and line 3 (**c**) in the WT, *astol1*(+/-) or *astol1*(+/+) background. Two plants each are shown. **(d-f)** Biomass of five-week-old WT, *astol1*(+/-) and the overexpression line 1 (**d**), line 2 (**e**) and line 3 (**f**) in the WT, *astol1*(+/-) or *astol1*(+/+) background. Scale bars, 10 cm. **(g-i)** Shoot S concentrations of five-week-old WT, *astol1*(+/-) and the overexpression line 1 (**g**), line 2 (**h**) and line 3 (**i**) in the WT, *astol1*(+/-) or *astol1*(+/+) background. **(j-l)** Root As concentrations of five-week-old WT, *astol1*(+/-) and the overexpression line 1 (**j**), line 2 (**k**) and line 3 (**l**) in the WT, *astol1*(+/-) or *astol1*(+/+) background. DW, dry weight. WT, wild type. *astol1*(+/-), *astol1* heterozygote. *astol1*(+/+), *astol1* homozygote. The letter P stands for the positive lines and the letter N for the null lines. Data in **d-l** are shown as means ± s.d., n = 3 biological replicates; each biological replicate represents an individual plant. Asterisks in **d-l** indicate significant differences by two-sided Student's t-test: *P<0.05, **P<0.01, ***P<0.001.

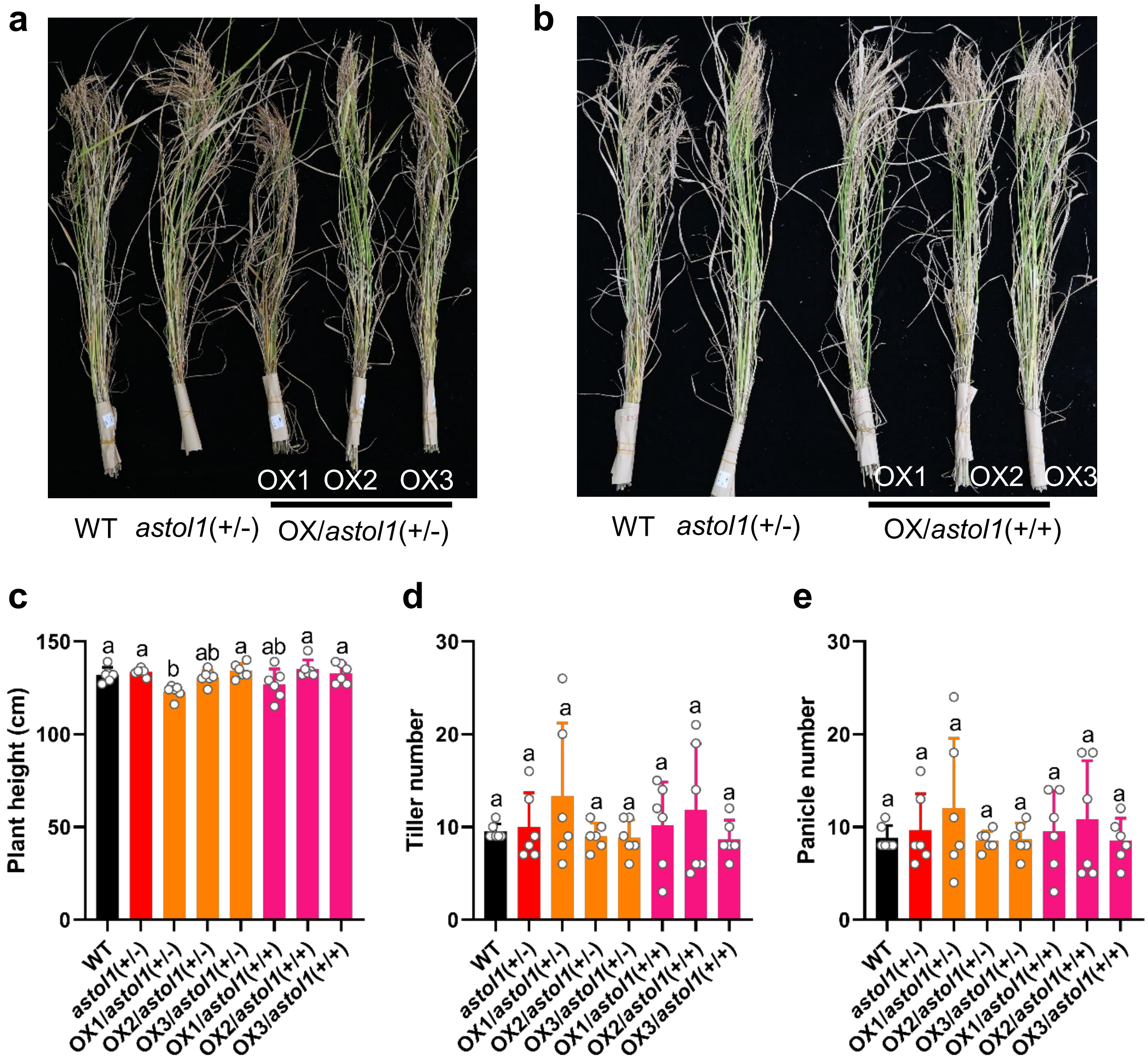

Supplementary Figure 21 Agronomic traits of the *OsASTOL1* overexpression lines in the *astol1*(+/-) or *astol1*(+/+) background.

(a) Wild-type rice, *astol1*(+/-) and three *OsASTOL1* overexpression lines in the *astol1*(+/-) background. **(b)** Wild-type rice, *astol1*(+/-) and three *OsASTOL1* overexpression lines in the *astol1*(+/+) background. Plants were grown in a paddy field (Lingshui) until maturity. At harvest, plant height (**c**), tiller number (**d**) and panicle number (**e**) were recorded. WT, wild type. *astol1*(+/-), *astol1* heterozygote. *astol1*(+/+), *astol1* homozygote. Data are shown as means ± s.d., $n = 6$ biological replicates; each biological replicate represents an individual plant. Different letters in **c-e** indicate significant differences ($P < 0.05$) using one-way ANOVA followed by Tukey's test.

Supplementary Table 1 Primers used in this study

Oligo name	Forward oligonucleotide (5'-3')	Reverse oligonucleotide (5'-3')
**dCAPS marker**		
ASTOL1-DdeI	ctcacgatgccggcctccctga	tcccagtatgagtccacagaacaca
**Sequencing for mutant site**		
ASTOL1-seq	cccgtgtctaagcctagaactcct	tcaggtccagtggtctcgtaatgg
**Vector constructs**		
ASTOL1/astol1 cDNA	atggccgtccaggttcaac	tcattcaaccaccatgttttcagc
ASTOL1/astol1 overexpression	ggatccccgggtaccatggccgtccagg	gagctctctagaactagttcattcaaccaccat
CRISPR target I	ggcaaatgggtgagaccatcgcca	aaactggcgatggtctcacccatt
CRISPR target II	gccgttggctcaatcagcacactc	aaacgagtgtgctgattgagccaa
ASTOL1 promotor-GUS	catgcggccgcttaattaaatcgcatggcatctc	gcggacctttgcacggcgcgccggtttgccagaag
ASTOL1/astol1-YFP	ggggacaagtttgtacaaaaaagcaggctatg gccgtccaggttcaac	ggggaccactttgtacaagaaagctgggtcttcaacca ccatgttttcagcttc
**CRISPR mutant identification**		
CRISPR target I seq	cttgttgaggagggagagcagat	accatccgaatgatccgtacataga
CRISPR target II seq	cccgtgtctaagcctagaactcctt	tcaggtccagtggtctcgtaatgg
**Overexpression identification**		
ASTOL1-OX	gccaaggatgtcaccgagttgatt	ttgtcttcgctgccagttcttctg
**Quantitative real-time PCR**		
OsASTOL1	cattgaaagaagggttgctggttgg	ggactggaagagcaccgacgaa
OsSultr1.1	cacggatctcatcggtgaagacaag	acttcctgtgctgctctgctctata
OsAPR1	tcgctcctcgccttcgtcaa	ggatcatggtcgtcgccaagtc
OsSiR	agaggttggctttgttggtgatgg	ccttcctgacgtgtgctgttcc
OsOAS-TL A	aagaagggctgctggttgggat	aagaggacagacgacaggtagcg
Os01g0978100 (OAS-TL)	tgacaaccctgccaacccaaag	ccacctgttccaattccaccaatga
Os01g0720700 (SAT)	cgacgatcctcggcaatgtcag	tgctggatgaaggaggtgtggt
Os05g0533500 (SAT)	gtctccatcctccaccacgttaca	cctcaccgttcttcctgccaatc
OsHistone H3	ttcaagaccgacctccgcttcc	gctggatgtccttgggcatgatg
**Enzyme activity assay**		
OsASTOL1-His	gaaggagatatacatatgatggccgtccaggtt caac	gtggtggtggtgctcgagttcaaccaccatgttttcagct tc
OsASTOL1ΔN87-His	gaaggagatatacatatgatgggtgagaccatc gcc	gtggtggtggtgctcgagttcaaccaccatgttttcagct tc

Continued Supplementary Table 1 Primers used in this study

Oligo name	Forward oligonucleotide (5'-3')	Reverse oligonucleotide (5’-3')
AtOAS-TL A cDNA	tgatagcgtgtgccatgtgactg	ggagaatgttcaagcctcgaaggt
AtOAS-TL A S102N	tgccagcttctatgaatactgagagaaga	tcttctctcagtattcatagaagctggca
AtOAS-TLA-His	gaaggagatatacatatgatggcctcgagaattgcta aagatg	gtggtggtggtgctcgagagcctcgaaggtcatggctt
OsASTOL1 S189A	ctccatggccatggagagga	tcctctccatggccatggag
OsASTOL1 S189C	ctccatgtgcatggagaggag	ctcctctccatgcacatggag
OsASTOL1 S189D	cctccatggacatggagagga	tcctctccatgtccatggagg
OsASTOL1 S189E	cctccatggagatggagaggag	ctcctctccatctccatggagg
OsASTOL1 S189F	cctccatgttcatggagaggag	ctcctctccatgaacatggagg
OsASTOL1 S189G	ctccatgggcatggagagga	tcctctccatgcccatggag
OsASTOL1 S189H	ctccatgcacatggagaggag	ctcctctccatgtgcatggag
OsASTOL1 S189I	cctccatgatcatggagaggag	ctcctctccatgatcatggagg
OsASTOL1 S189K	cctccatgaagatggagaggag	ctcctctccatcttcatggagg
OsASTOL1 S189L	cctccatgctcatggagagga	tcctctccatgagcatggagg
OsASTOL1 S189M	cctccatgatgatggagaggag	ctcctctccatcatcatggagg
OsASTOL1 S189P	ctccatgcccatggagagga	tcctctccatgggcatggag
OsASTOL1 S189Q	cctccatgcagatggagagga	tcctctccatctgcatggagg
OsASTOL1 S189R	cctccatgaggatggagagga	tcctctccatcctcatggagg
OsASTOL1 S189T	cctccatgaccatggagagga	tcctctccatggtcatggagg
OsASTOL1 S189V	cctccatggtcatggagagga	tcctctccatgaccatggagg
OsASTOL1 S189W	cctccatgtggatggagagga	tcctctccatccacatggagg
OsASTOL1 S189Y	cctccatgtacatggagaggag	ctcctctccatgtacatggagg
**Pull-down assay**		
OsASTOL1ΔN87	gaaggagatatacatatgggtgagaccatcgccaa	gcaagcttgtcgacggagctctcattcaaccaccatgttt tcagct
AtOAS-TLA	gaaggagatatacatatggcctcgagaattgctaaa ga	gcaagcttgtcgacggagctctcaagcctcgaaggtca tgg
S-OsASTOL1ΔN87	tggtgccacgcggttccatggctatgggtgagacca tcgccaa	gcaagcttgtcgacggagctctcattcaaccaccatgttt tcagct
S-AtOAS-TLA	tggtgccacgcggttccatggctatggcctcgagaa ttgctaaagatg	gcaagcttgtcgacggagctcttatatgatgtaatctgac cattccgagatg

Supplementary Table 2 Prediction of chloroplast transit peptides (cTP) in OsASTOL1 protein sequence and the location of potential cTP cleavage site (CS).

The OsASTOL1 full length protein sequence was used to predict the presence of the chloroplast transit peptides (cTP) and the location of potential cTP cleavage site (CS) in ChloroP 1.1 (Server website: http://www.cbs.dtu.dk/services/ChloroP/). Score is the output score from the second step network. The prediction cTP/no cTP is based solely on this score. "Y" means that the sequence is predicted to contain a cTP; CS-score is the MEME scoring matrix score for the suggested cleavage site. cTP-length is the predicted length of the presequence.

Name	Length	Score	cTP	CS-score	cTP-length
OsASTOL1	408	0.523	Y	7.602	76

Supplementary Table 3 The kinetic properties of OAS-TL proteins binding to OAS or AtSAT5C10 peptide.

The affinity (K_d value) of recombinant His-tagged OAS-TL proteins binding to OAS or AtSAT5C10 peptide were measured by microscale thermophoresis (MST) analysis with the standard procedure. N.D., Not determined. *n* = 3.

	OAS	AtSAT5C10 peptide
OsASTOL1	1.47±2.42 nM	468.18±322.79 nM
OsASTOL1^{S189N}	N.D.	345.44±296.02 nM
AtOAS-TLA	13.13±4.83 nM	34.27±10.53 nM
AtOAS-TLA^{S102N}	N.D.	36.48±15.62 nM

Supplementary Table 4 The concentrations (nmol/g FW) of serine and sulfur-related metabolites in rice. The wild type (WT) and *astol1*(+/-) mutant were grown in ½ Kimura B nutrient solution for 4 weeks. Data represent means ± SD (n = 5 for sulfate, n = 3 for other metabolites; each biological replicate represents one individual plant). Abbreviations of metabolites: SO_4^{2-}, sulfate; Ser, serine; OAS, *O*-acetylserine; Cys, cysteine; Cyst, cystathionine; GSH, glutathione; Hcy, homocysteine; Met, methionine; SAH, *S*-adenosylhomocysteine; SAM, *S*-adenosylmethionine; MTA, methylthioadenosine.

nmol/g FW	Roots		Leaves	
	WT	*astol1*(+/-)	WT	*astol1*(+/-)
SO_4^{2-}	10668.4±525.8	14325.5±1141.8	8161.2±570.7	36863.5±2170.9
Ser	11.82±0.26	10.33±0.75	18.91±1.62	13.64±0.95
OAS	4.18±1.16	29.77±1.47	20.39±3.15	128.03±4.00
Cys	1.83±0.34	14.49±0.51	16.99±3.61	83.94±5.83
GSH	7.19±0.10	8.83±0.25	184.6±9.15	525.6±105.0
Cyst	0.002±0.001	0.054±0.008	0.010±0.005	0.178±0.012
Hcy	60.58±6.74	98.54±2.02	10.97±3.65	16.67±2.23
Met	9.53±0.79	10.13±0.47	8.99±0.35	9.96±1.07
SAM	6.48±0.44	7.71±0.24	9.95±1.40	15.12±0.94
MTA	12.96±1.14	21.21±0.70	8.76±0.75	13.27±1.01
SAH	0.12±0.02	0.17±0.03	0.20±0.01	0.19±0.04
