# Supplementary material for: A molecular switch in sulfur metabolism to reduce arsenic and enrich selenium in rice grain
Source: Nat Commun. 2021 Mar 2;12:1392. doi: 10.1038/s41467-021-21282-5 (PMC7925690; doi:10.1038/s41467-021-21282-5)
Supplement: Supplementary file 3 — Reporting Summary [file 41467_2021_21282_MOESM3_ESM.pdf]

Corresponding author(s): Fang-Jie Zhao

Last updated by author(s): Jan 15, 2021

Reporting Summary

Nature Research wishes to improve the reproducibility of the work that we publish. This form provides structure for consistency and transparency in reporting. For further information on Nature Research policies, see our Editorial Policies and the Editorial Policy Checklist.

Statistics

For all statistical analyses, confirm that the following items are present in the figure legend, table legend, main text, or Methods section.

n/a	Confirmed	
☐	☒	The exact sample size (n) for each experimental group/condition, given as a discrete number and unit of measurement
☐	☒	A statement on whether measurements were taken from distinct samples or whether the same sample was measured repeatedly
☐	☒	The statistical test(s) used AND whether they are one- or two-sided<br>*Only common tests should be described solely by name; describe more complex techniques in the Methods section.*
☒	☐	A description of all covariates tested
☐	☒	A description of any assumptions or corrections, such as tests of normality and adjustment for multiple comparisons
☐	☒	A full description of the statistical parameters including central tendency (e.g. means) or other basic estimates (e.g. regression coefficient) AND variation (e.g. standard deviation) or associated estimates of uncertainty (e.g. confidence intervals)
☐	☒	For null hypothesis testing, the test statistic (e.g. F, t, r) with confidence intervals, effect sizes, degrees of freedom and P value noted<br>*Give P values as exact values whenever suitable.*
☒	☐	For Bayesian analysis, information on the choice of priors and Markov chain Monte Carlo settings
☒	☐	For hierarchical and complex designs, identification of the appropriate level for tests and full reporting of outcomes
☒	☐	Estimates of effect sizes (e.g. Cohen's d, Pearson's r), indicating how they were calculated

Our web collection on statistics for biologists contains articles on many of the points above.

Software and code

Policy information about availability of computer code

Data collection	ICP-MS data were collected by Perkin Elmer NexION software (version 1.5). HPLC data were collected by Chromera software (version 2.1). IC data were collected by Chromeleon 7 software. Q-PCR data were collected by Bio-Rad CFX Manager 3.1 software. SDS-PAGE gels and blots were detected on Bio-Rad Image Lab software (version 5.2). Microscale thermophoresis data were collected by MO. Control software (version 1.6.1). Absorbence data were collected by UVProbe software (version 2.42). Image data were collected by ZEISS ZEN 2.3 software and OLYMPUS Stream Image Analysis Software (version 2.4).
Data analysis	Resequencing data were analyzed by BWA software (version 1.1) and GATK software (version 4). Microscale thermophoresis data were analyzed by MO. Affinity Analysis software (version 2.3). The blots were quantified by Image J software (version 1.52). Multiple sequence alignments were performed by DNAMAN software (version 7). Phylogenetic tree was constructed using MEGA 4.0 software. Protein structure modeling was performed by PyMOL sofeware (version 1.8). Microsoft Excel 2016, Origin 2019 and GraphPad Prism 8.0 were used for data analysis and statistics.

For manuscripts utilizing custom algorithms or software that are central to the research but not yet described in published literature, software must be made available to editors and reviewers. We strongly encourage code deposition in a community repository (e.g. GitHub). See the Nature Research guidelines for submitting code & software for further information.

Data

Policy information about availability of data

All manuscripts must include a data availability statement. This statement should provide the following information, where applicable:

- Accession codes, unique identifiers, or web links for publicly available datasets
- A list of figures that have associated raw data
- A description of any restrictions on data availability

The authors declare that all data supporting the findings of this work are available within the paper and its Supplementary Information files. The source data for all Figures and Supplementary Figures and Tables are provided as a Source Data file.

Field-specific reporting

Please select the one below that is the best fit for your research. If you are not sure, read the appropriate sections before making your selection.

[x] Life sciences [ ] Behavioural & social sciences [ ] Ecological, evolutionary & environmental sciences

For a reference copy of the document with all sections, see nature.com/documents/nr-reporting-summary-flat.pdf

Life sciences study design

All studies must disclose on these points even when the disclosure is negative.

Sample size	The sample size for each experiment is described in the Figure and Table legends and is mainly based on our past experience performing similar experiments.
Data exclusions	No data were excluded from the analysis.
Replication	At least three replicates were performed for related experiments, and described in the Figure legends.
Randomization	For all experiments, we used randomization to allocate different genotypes subjected to different treatments.
Blinding	For all experiments, we analyzed the results in different genotypes subjected to different treatments and therefore, blinding was not possible for us.

Reporting for specific materials, systems and methods

We require information from authors about some types of materials, experimental systems and methods used in many studies. Here, indicate whether each material, system or method listed is relevant to your study. If you are not sure if a list item applies to your research, read the appropriate section before selecting a response.

Materials & experimental systems

n/a	Involved in the study	
[ ]	[x]	Antibodies
[x]	[ ]	Eukaryotic cell lines
[x]	[ ]	Palaeontology and archaeology
[x]	[ ]	Animals and other organisms
[x]	[ ]	Human research participants
[x]	[ ]	Clinical data
[x]	[ ]	Dual use research of concern

Methods

n/a	Involved in the study	
[x]	[ ]	ChIP-seq
[x]	[ ]	Flow cytometry
[x]	[ ]	MRI-based neuroimaging

Antibodies

Antibodies used	A rabbit anti-OAS-TL A antibody, a rabbit anti-RbcL antibody (AS03037, Agrisera), a rabbit anti-S-tag antibody (101290-T38, Sino Bio), a mouse anti-His-tag antibody (AF5060, Beyotime), a HPR-conjugated goat anti-mouse IgG (AT0098, CMCTAG) and a HPR-conjugated goat anti-rabbit IgG (AT0097, CMCTAG)were used in this study.
Validation	anti-OAS-TLA antibody (against full-length Arabidopsis cytosolic OAS-TL A) was generated in a previous study reported in this reference. https://www.sciencedirect.com/science/article/pii/S0378111900002614<br>Information of anti-RbcL antibody validation can be found at the product website. https://www.agrisera.com/en/artiklar/-rbcl-rubisco-large-subunit-form-i-rabbit.html<br>Information of anti-S tag antibody validation can be found at the product website. https://tw.sinobiological.com/antibodies/s-tag-101290-t38<br>Information of anti-His tag antibody validation can be found at the product website. https://www.beyotime.com/product/

AF5060.htm
Information of HPR-conjugated goat anti-rabbit IgG antibody validation can be found at the product website. http://www.cmctag.com/products/Goat-Anti-Rabbit-IgG-H-L-HRP-AT0097.html
Information of HPR-conjugated goat anti-mouse IgG antibody validation can be found at the product website. http://www.cmctag.com/products/Goat-Anti-Mouse-IgG-H-L-HRP-AT0098.html
